# Oxidative Stress in Women Treated with Atosiban for Impending Preterm Birth

**DOI:** 10.1155/2018/3919106

**Published:** 2018-12-02

**Authors:** Mariusz Grzesiak, Zuzanna Gaj, Rafał Kocyłowski, Joanna Suliburska, Przemysław Oszukowski, Wojciech Horzelski, Constantin von Kaisenberg, Maciej Banach

**Affiliations:** ^1^Department of Obstetrics, Perinatology and Gynecology, Polish Mother's Memorial Hospital-Research Institute, Lodz 93-338, Poland; ^2^Scientific Laboratory of the Center of Medical Laboratory Diagnostics and Screening, Polish Mother's Memorial Hospital-Research Institute, Lodz 93-338, Poland; ^3^PreMediCare New Med Medical Centre, Poznan 61-693, Poland; ^4^Institute of Human Nutrition and Dietetics, Poznan University of Life Sciences, Poznan 60-624, Poland; ^5^Department of Obstetrics and Perinatology, Medical University of Lodz, Zgierz 95-100, Poland; ^6^Faculty of Mathematics and Computer Science, University of Lodz, Lodz 90-238, Poland; ^7^Department of Obstetrics and Gynecology, Hannover Medical School, Hannover 30625, Germany; ^8^Department of Hypertension, Medical University of Lodz, Lodz 90-549, Poland

## Abstract

Preterm birth is defined as delivery before 37 completed weeks of pregnancy, and it is the leading cause of neonatal morbidity and mortality. Oxidative stress is recognized as an important factor in the pathogenesis of premature labor. We conducted this analysis to investigate the safety of administration of the tocolytic drug Atosiban—a reversible, competitive antagonist of the oxytocin receptor in the treatment of preterm birth and its impact on the level of oxidative stress in pregnant women after 48 hours of tocolytic treatment. This prospective study was conducted between March 2016 and August 2017 at the Obstetric Clinic of the Polish Mother's Memorial Hospital Research Institute. Total oxidant status (TOS), total antioxidant status (TAS), and oxidative stress index (OSI) values as well as 3-nitrotyrosine, carbonyl, and thiol group levels were measured using an ELISA test in serum and plasma of 56 pregnant women before and after 48 hours of continuous administration of Atosiban. We found that TAS levels decreased almost twice after the 48-hour drug administration (0.936 ± 0.360 mmol/L vs. 0.582 ± 0.305 mmol/L, *P* < 0.001) while TOS increased from 18.217 ± 16.093 *μ*mol/L to 30.442 ± 30.578 *μ*mol/L (*P* < 0.001). We also found a significant increase in OSI index—almost a threefold increase from 0.022 ± 0.022 to 0.075 ± 0.085, *P* < 0.001. In addition, statistically significant differences in the level of carbonyl groups were found. It increased from 65.358 ± 31.332 *μ*mol/L to 97.982 ± 38.047 *μ*mol/L (*P* < 0.001), which indicates increased oxidation of plasma proteins. Furthermore, patients who gave birth prematurely had higher levels of TOS after a 48-hour drug administration than the second group with labor after 37 weeks of pregnancy (42.803 ± 34.683 *μ*mol/L vs. 25.792 ± 27.821 *μ*mol/L, *P* < 0.031). The obtained results clearly indicate that pregnant women during tocolytic treatment with Atosiban are in a state of increased oxidative stress and occurrence of preterm birth can be associated with this phenomenon. This trial is registered with NCT03570294.

## 1. Introduction

Oxidative stress is defined as an imbalance in the production of reactive oxygen species and the capacity of antioxidant defenses. A moderate level of oxidative stress was found as normal in pregnancy [1–3]. However, when increased production of reactive nitrogen and oxygen species (RNS and ROS, respectively) exceeds the mother's antioxidant potential, it can negatively affect the well-being of both the mother and the fetus, leading to complications in pregnancy, as well as adverse pregnancy outcomes such as spontaneous preterm birth and preterm premature rupture of membranes [4–9]. The modifications caused by RNS and ROS result in new functional groups such as hydroxyl or carbonyl groups, leading to fragmentation of protein, formation of protein-protein cross-linkages, disruption of the tertiary structure, and a loss of functional activity [1, 3].

Preterm labor is defined as delivery before 37 completed weeks of gestation and can affect 5–13% of all pregnancies in high-income countries. It is the important cause of neonatal morbidity and mortality because it can be responsible for even 50% of neonatal morbidity and 50–75% of neonatal mortality worldwide [10]. The most common phenotype of preterm labor is spontaneous preterm birth of unknown etiology; however, an uncompensated increase in oxidative stress is considered as one of the important reasons for premature birth [2, 11]. The current evidence suggests that the administration of tocolytic drugs can delay preterm labor for 48 hours, thus allowing administration of corticosteroids to stimulate production of surfactant in fetal lungs which can improve neonatal outcome [12, 13].

Atosiban (1-(3-mercaptopropanoic acid)-2-(O-ethyl-D-tyrosine)-4-L-threonine-8-L-ornithine-oxytocin) is a reversible, competitive antagonist of the oxytocin receptor (OTR) and can decrease contractions and improve uterine receptivity due to its ability to reduce intracytoplasmic calcium release and downregulate prostaglandin synthesis [14–16]. Atosiban is licensed for clinical use in case of impending preterm birth and is commonly used in clinical practice in Europe because of its low side effect profile [17–21]. While a role of Atosiban in the modulation of myometrial contractility is well-described, its effect on many other functions is not so well known. Recent publications presented new data about its role in elevated cardiac oxidative stress in newborn rats from mothers treated with Atosiban and activation of the inflammatory pathway in the amnion which may reduce its potency as a tocolytic drug [22–24].

We conducted this analysis to investigate the safety of administration of Atosiban in the treatment of preterm labor and its impact on the level of oxidative stress after 48 hours of tocolytic treatment. To the best of our knowledge, this is the first analysis concerning the association of Atosiban treatment on the total oxidant and antioxidant status in pregnant women suffering from threatened premature birth.

## 2. Material and Methods

### 2.1. Patients

This study was conducted between March 2016 and August 2017 at the Obstetric Clinic of the Polish Mother's Memorial Hospital Research Institute which is a tertiary referral hospital. The study protocol conformed to the principles of the Helsinki Declaration and was approved by the local Research Ethical Committee (No. 34/2014). Informed consent was obtained from all women before inclusion. This study was retrospectively registered with ClinicalTrials.gov on 26 June 2018. Sixty-four pregnant women receiving prenatal care due to the risk of premature birth who met the inclusion criteria were enrolled in the study. The study also involved 8 patients with twin pregnancies. The average results for tested biochemical parameters in the group with twin pregnancy were very similar to the results of the group with a single pregnancy; however, it was decided to exclude them from the analysis to eliminate confounding factors. The final analysis comprised 56 women. Inclusion criteria were as follows: patients between 24 and 35 weeks' gestation with intact membranes and showing evidence of premature labor. The last menstrual date or ultrasonographic measurements from the first trimester were used to determine the gestational age. The preterm labor was diagnosed as painful, regular, and persistent uterine contractions (no less than a 30-second duration and at least four in an hour) before the 37th week of gestation associated with cervical changes (a cervical dilation of 1 to 3 cm) and/or effacement (≥50%). Exclusion criteria included acute fetal distress and other conditions requiring immediate delivery (eclampsia and severe preeclampsia, abruptio placenta, and placenta previa) as well as chorioamnionitis, vaginal bleeding, intrauterine growth restriction, and fetal congenital malformations. The use of any tocolytic drugs during pregnancy before admission to the hospital also met the exclusion criteria. The patients with circulatory system diseases (e.g., heart defects and hypertension), symptoms of infection and other diseases that may increase oxidative stress, and any other specific maternal contraindication for Atosiban treatment were also excluded. According to our data, none of the subjects in the study were smokers or were using specific antioxidant supplementation.

### 2.2. Tocolytic Treatment

Atosiban medication was administered after a particular patient evaluation, in accordance with the drug characteristic medical protocol. The initial dose of Atosiban (Tractocile, Ferring Pharmaceuticals A/S, Copenhagen, Denmark) was given as a single intravenous bolus dose (6.75 mg in 0.9 mL isotonic sodium chloride solution). This was followed immediately by intravenous infusion of 300 *μ*g/min of Atosiban in 5% glucose for 3 hours and then 100 *μ*g/min for up to 48 hours. Maternal steroid therapy was started right after admission to the hospital. According to the most recent recommendations from The American College of Obstetricians and Gynecologists, patients were given four 6 mg doses of dexamethasone (Dexaven, Jelfa, Poland) administered intramuscularly every 12 hours [25].

### 2.3. Blood Sampling

Venous blood samples from a forearm antecubital vein were taken before and after 48 hours of continuous administration of tocolytic therapy with Atosiban. Blood samples were collected into serum-separating tubes and in tubes containing EDTA (for plasma) and immediately stored at 4°C. The plasma was then separated from the cells by centrifugation at 1500× g for 20 min, at 4°C. In order to obtain serum, the blood was centrifuged at 2500× g, at 4°C for 10 minutes. The serum and plasma samples were taken for the measurement of total oxidant status (TOS), total antioxidant status (TAS), level of 3-nitrotyrosine (3-NT), and carbonyl and thiol groups which were stored at −70°C in aliquots for subsequent biochemical analysis and processed within two months. They were thawed at room temperature only once at the time of analysis. All measurements were performed in duplicate.

### 2.4. Measurement of Total Oxidant and Antioxidant Status (TOS and TAS) in Serum

Total oxidant status and total antioxidant status values were measured by colorimetric assay according to a method developed by Erel [26, 27]. The TOS Rel Assay Diagnostics kit (Rel Assay, Gaziantep, Turkey) was used to determine the levels of the total oxidant status according to the manufacturer's instructions. This method is based on the iron-mediated reactions of apparently stable products (hydroperoxides) of the lipid peroxidation process. The oxidation of ferrous ion to ferric ion in the presence of various oxidant species in serum was measured using xylenol orange. The assay was calibrated with hydrogen peroxide, and the results are expressed in terms of micromolar hydrogen peroxide equivalent per liter (*μ*mol H_2_O_2_ equiv./L). The coefficients of variation values were found less than 10%. TAS was measured by determination of the total nonenzymatic plasma antioxidant capacity using the TAS Rel Assay Diagnostics kit (Rel Assay, Gaziantep, Turkey). In this method, 2,2′-azino-di-(3-ethylbenzthiazoline sulfonate) radical cation (ABTS^∗^^+^, ^∗^ is an unpaired electron) produced by the hydroxyl radical is used as an indicator of antioxidant activity. The reaction was calibrated with Trolox (a water-soluble analogue of vitamin E, 6-hydroxy-2.5.7.8-tetramethylchroman-2-carboxylic acid), and the results were expressed as mmol Trolox equiv./L. The coefficients of variation values were found less than 10%.

### 2.5. Oxidative Stress Index (OSI) Determination

OSI was determined as a TOS to TAS ratio (the resulting unit of TAS was changed to *μ*mol/L) and was calculated as follows: OSI (arbitrary unit) = TOS, *μ*mol H_2_O_2_ equiv./L/TAS, and *μ*mol Trolox equiv./L [23].

### 2.6. Estimation of 3-Nitrotyrosine

The competitive 3-nitrotyrosine (3-NT) ELISA Kit was used to determine protein peroxidation in plasma (OxiSelect™ Nitrotyrosine ELISA Kit, Cell Biolabs Inc., San Diego, USA). 3-NT is a product of posttranslational modification of protein tyrosine caused by peroxynitrite, and this modification can result in changes in protein structure, function, and catalytic activity [28]. The absorbance was read at 450 nm against 620 nm as a reference with an ELISA reader iMark™ (Bio-Rad USA). The protein nitrotyrosine content was determined by comparing with a standard curve that was prepared from predetermined nitrated BSA standards (2.7 mole of nitrotyrosine per 1 mole of BSA). The detection limit was 20 nM.

### 2.7. Estimation of Protein Carbonyl Groups

The measurement of protein carbonyl content is considered a reliable marker of oxidative protein damage due to its long-lasting stability [28]. Protein carbonyls were measured as a parameter of oxidative stress in plasma after derivatization with 2,4-dinitrophenylhydrazine (DNPH) with the Carbonyl Protein ELISA Kit (Immundiagnostik AG, Bensheim, Germany) according to the instructions supplied by the manufacturer. The absorbance was read at the wavelength of 450 nm against 620 nm as a reference with an ELISA reader iMark™ (Bio-Rad). The intra- and interassay coefficients of variability were found less than 6.5% and 6.2%, respectively.

### 2.8. Estimation of Thiol Groups

Thiol protein (sulfhydryl) status (glutathione, protein-bound, and free SH groups) was measured in plasma by enzyme-linked immunosorbent assay according to the manual instructions (Immundiagnostik AG, Bensheim, Germany) using a modification of Ellman's method. Serum protein thiols react with oxygen-containing free radicals to form disulfides; therefore, they are a good and direct measure of the *in vivo* redox status in human. Absorbance was measured at 412 nm against a reagent blank using an ELISA reader Synergy H1 (BioTek, Vermont, USA).

### 2.9. Statistical Analysis

Statistical analyses were performed using MedCalc for Windows, version 15.1 (MedCalc Software, Ostend, Belgium). The normality of data was tested using the D'Agostino-Pearson test. The results were presented as means with standard deviation (SD) or as median with interquartile range (25th–75th percentiles) for nonnormally distributed variables (gestational age at baseline, parity, gravidity, and gestational age at delivery). The baseline characteristics were compared depending on the assumptions using Student's paired *t*-test for normally distributed data or Wilcoxon's test for paired samples for nonnormally distributed variables. Analysis of variance (ANOVA) tests were used to examine the relationship between time of labor and levels of markers of oxidative stress. *P* values less than 0.05 were considered statistically significant.

## 3. Results

Sociodemographic, obstetric, and neonatal characteristics of the 56 women included in the study are presented in Table 1. The mean age of the examined women was 30.9 ± 5.4 years, 50% of patients (28/56) were nulligravida, and 64% of women (36/56) were nulliparous. Atosiban was administered on average at 30 ± 2.9 weeks of pregnancy. Looking at the results of our studies, 96% of the women treated with Atosiban were still pregnant one week after treatment (54 out of 56) and the longest period from administration of the drug to delivery was 14 weeks (mean 6.5 ± 3.5 weeks). The patients gave birth on average at 36.6 ± 3.1 weeks of pregnancy, and 60.3% of pregnant women delivered by cesarean section, while 39.7% gave birth naturally. Despite the fact that patients gave birth on average at 36.6 weeks of pregnancy which could suggest preterm birth, most of pregnant women in our study (35/53, 66%) had labored after 37 completed weeks of pregnancy which confirms the tocolytic effectiveness of Atosiban. The mean birth weight of the newborns was 2884.8 ± 748.8 g, and they received an average of 8.7 ± 2 points on the Apgar scale. Most children (82.3%, 42/51) left the hospital during the first week of life in good condition; two children born before the 27th week of pregnancy died due to extreme prematurity and low birth weight (600 and 720 g, respectively). Only three children born between 28 and 31 weeks of pregnancy, due to complications related to prematurity, had to spend more than 4 weeks in the neonatal ward. Three children were lost to follow-up because their mothers gave birth in another hospital.

**Figure 1:**
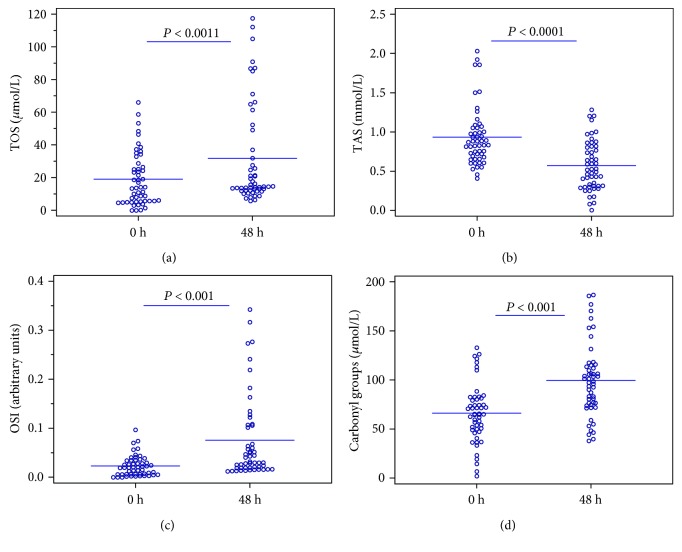
Analyses of total oxidative status (a), total antioxidant status (b), OSI index (c), and carbonyl group level (d) before and after a 48-hour treatment with Atosiban. The horizontal lines represent the mean values.

According to biochemical markers, both TOS and protein carbonyls as well as OSI index were increased in serum collected after 48 h therapy when compared to the serum before administration of Atosiban (Table 2). It was also shown that the total serum antioxidative potential decreased almost twice after a 48-hour drug administration from an average of 0.936 ± 0.360 mmol/L to 0.582 ± 0.305 mmol/L (*P* < 0.001). On the other hand, the total oxidation potential increased from the mean value of 18.217 ± 16.093 *μ*mol/L in the samples taken before drug administration to the value of 30.442 ± 30.578 *μ*mol/L (*P* < 0.001) in samples after 2 days of continuous administration of Atosiban. The obtained results as well as a significant increase in OSI index which is a cumulative marker of both oxidative and antioxidative power (an over threefold increase from 0.022 ± 0.022 to 0.075 ± 0.085, *P* < 0.001) clearly indicate a higher level of oxidative stress after the treatment with Atosiban and a significant reduction in the antioxidative capacity of the investigated serum. Analyses of total oxidative status, total antioxidant status, and OSI index before and after treatment are presented in Figures 1(a)–1(c). In addition, statistically significant differences in the level of carbonyl groups in the tested plasma were found (Figure 1(d)). It increased from 65.358 ± 31.322 *μ*mol/L to 97.982 ± 38.047 *μ*mol/L (*P* < 0.001), which indicates increased oxidation of serum proteins and their damage. On the other hand, we did not observe statistically significant differences in the level of thiol groups. 3-NT concentrations, an indicator of protein peroxidation and oxidative stress, were also not significantly different between the groups (*P* = 0.230); however, the higher concentration of this marker was observed in the serum collected after treatment (40.7 ± 44.2 nM vs. 51.4 ± 44.1 nM, respectively).

An additional analysis of the levels of oxidative stress markers was carried out in groups of women with preterm delivery (*n* = 18) and with labor after 37 weeks of pregnancy (*n* = 35). Atosiban was administered at a very similar period of pregnancy for both groups, on average at 30.1 ± 3.7 weeks of pregnancy in the group with preterm delivery and at 30.2 ± 2.5 weeks of pregnancy in the other group with labor after 37 weeks of pregnancy. Patients who gave birth prematurely had higher levels of TOS after a 48-hour drug administration than the second group (42.803 ± 34.683 *μ*mol/L vs. 25.792 ± 27.821 *μ*mol/L, *P* < 0.031) (Table 3). Despite the lack of statistically significant differences for other markers, we also observed an increase in oxidative stress parameters (TOS and carbonyl groups) and a reduction in the antioxidative capacity (TAS) in the compared groups before and after 48 hours of therapy with Atosiban (Table 3).

## 4. Discussion

In this present study, we demonstrated, for the first time, a higher oxidative status (reflected by increased levels of TOS, OSI index, and carbonyl group level) and lower antioxidant capacity caused by oxidative stress in the serum of pregnant women after tocolytic treatment. Furthermore, patients who gave birth prematurely had higher levels of TOS after a 48-hour administration of Atosiban than the second group with labor after 37 weeks of pregnancy, which might indicate the influence of increased oxidative stress on preterm labor.

This study was conducted to investigate the safety of administration of Atosiban in the treatment of preterm labor. Atosiban has proved to be a very effective short-term tocolytic drug which is consistent with the Polish recommendations suggesting its use as the first-line drug in the treatment of preterm labor [29]. Also, our present observation did not confirm the reports about the adverse impact of Atosiban on the newborns [30]. Simsek et al. [23] indicated that blockage of the oxytocin receptor may be connected with increased fetal mortality and morbidity because of the elevated oxidative stress observed in the heart of the newborn rats. However, we have not found such negative effects and the vast majority of neonates in our study were born in good condition. A detailed evaluation of placental and fetal circulation with assessment of cardiac hemodynamic function during 48 hours of administration of Atosiban in pregnant women carried out in our previous studies showed that hemodynamic cardiac activity in fetuses remained unaffected after tocolytic treatment [31, 32]. It can be explained by the fact that Atosiban crosses the placenta with an average fetal versus maternal ratio of 0.124 and its concentrations do not appear to accumulate in the fetus [33].

Nevertheless, we observed that treated women were in a state of increased oxidative stress after a 48-hour administration of Atosiban. Unfortunately, very little is known about the possible involvement of tocolytic therapy in oxidative stress damage. We demonstrated that serum and plasma samples showed higher level of TOS and carbonyl groups as well as a significant increase in OSI index after tocolytic treatment. The statistically significant differences in TAS levels can also suggest that systemic antioxidant defense mechanisms are exhausted during treatment due to increased oxidative stress. A very similar pattern was observed in the studies conducted by Aycicek et al. [34] on active and passive maternal smoking. Cigarette smoke is a well-known risk factor of adverse pregnancy outcomes, and exposure to this external stressor agent can cause important alterations in oxidant and antioxidant balance and increase potent oxidative stress [3, 35]. The increased level of TOS and OSI index as well as lower antioxidant capacity after tocolytic treatment with Atosiban were comparable to those observed in maternal serum from the active smoker group [34]. Similarly, Argüelles et al. [36] found that smoking mothers and their newborns had a higher concentration of the carbonyl group and lipid peroxides and lower total antioxidant capacity in serum and umbilical cord blood than nonsmoking mothers and their newborns from the control group.

A relationship between administration of Atosiban and increase in oxidative stress parameters was also observed in an animal research study conducted by Simsek et al. [23]. They found higher levels of oxidative stress markers (TOS and OSI index) as well as a decrease in TAS level in the plasma of newborn rats in the Atosiban-treated group when compared to the control group. This observation can be an indirect evidence of Atosiban's involvement in a process of increasing levels of oxidative stress because in this study model, the pregnant female rats were treated only with this tocolytic drug [23]. According to Martin et al. [37], high oxidative stress level in mothers could be responsible for the high oxidative stress level observed in newborns by direct blood exchange in the placenta. Moreover, also Argüelles et al. [36] found a correlation between the oxidative state of the mother and the neonate, and importantly, they observed that a higher level of maternal oxidative stress corresponds to an even higher oxidative stress in newborn umbilical cord blood.

Several studies have implicated the role of oxidative stress in preterm birth [2, 3, 5, 8, 38–40]. A high level of systemic oxidative stress in the mother can cause placental dysfunctions or other damages leading to preterm labor [3, 8, 9, 37, 38, 41]. Most of pregnant women in our study (66%) had labored after 37 completed weeks of pregnancy; however, we found a statistically significant association between levels of TOS and preterm birth. Patients who gave birth prematurely had higher levels of TOS after a 48-hour administration of Atosiban than the second group with labor after 37 weeks of pregnancy, which might indicate the influence of increased oxidative stress on preterm labor. This is consistent with many studies measuring RNS and ROS or biomarkers of oxidative stress which reported higher levels of these products and lower levels for antioxidants for preterm specimens compared to term birth specimens [5, 9, 39, 42, 43].

The limitation of this study was the relatively small sample size used and a nonrandomized study design. Moreover, all treated patients received corticosteroids which could potentially influence the changes in oxidative stress markers. However, the main purpose of using tocololytics is to delay delivery to allow the administration of a complete course of antepartum corticosteroids so this factor could not be ruled out to examine their effect independently of each other. We also could not exclude the influence on women such factors as pain, uterine contractions, and stress resulting from the threat of premature delivery. Nevertheless, the imbalance in the levels of oxidative stress biomarkers corresponded well with some previous studies regarding complications of pregnancy and results of animal studies on Atosiban and oxidative stress. Another limitation is the lack of evaluation of dietary intake of antioxidants; however, according to our data, none of the subjects in the study were smokers or were using specific antioxidant supplementation.

## 5. Conclusions

In conclusion, our results can indicate that tocolytic treatment with Atosiban is associated with elevation of oxidative stress markers after a 48 h administration. This effect of Atosiban may reduce its potency as a tocolytic agent and therefore should be considered with respect to its clinical use, especially because of its connection with the occurrence of premature birth. Further studies involving a large sample size should be undertaken to examine the strength of this observation and can clearly give evidence to the Atosiban effect in addition to steroids on this phenomenon. It is also worth considering whether the addition of antioxidant therapy can be applied in women treated with oxytocin antagonist and whether it can increase its effectiveness.

## Figures and Tables

**Table 1:** Sociodemographic, obstetric, and clinical characteristics of study participants.

Variables	
*Mothers (n* = 56)	
Age (years)^a^	31 ± 5.3
Gestation age at baseline (weeks)^b^	30 (28–32)
Parity^b^	1.59 (1–2)
Gravidity^b^	1 (1–2)
Gestation age at delivery (weeks)^b^	37 (36–39)
*Children (n* = 53)	
Male/female^c^	60.4/39.6
Birth weight (g)^a^	2884.8 ± 748.8
Apgar scale^b^	10 (9–10)
Days of hospitalization^a^	8.1 ± 10.1

^a^Values are means and standard deviation (SD), ^b^values are median and interquartile range (25th–75th percentiles), and ^c^values are percentage.

**Table 2:** Oxidative and antioxidative parameters in plasma and serum before and after 48 hours of continuous administration of tocolytic therapy with Atosiban.

Treatment with Atosiban			
Variable	0 hours	48 hours	*P* value
TOS (*μ*mol/L)	18.217 ± 16.093	30.442 ± 30.578	**0.0011** ^a^
TAS (mmol/L)	0.936 ± 0.360	0.582 ± 0.305	**<0.0001** ^a^
OSI (arbitrary units)	0.022 ± 0.022	0.115 ± 0.395	**<0.001** ^a^
Carbonyl groups (*μ*mol/L)	65.358 ± 31.322	97.982 ± 38.047	**<0.001** ^b^
Thiol groups (*μ*mol/L)	512.023 ± 121.162	516.240 ± 111.886	0.806^a^
3-Nitrotyrosine (nM)	40.8 ± 45.7	50.5 ± 44.7	0.210^a^

Data are presented as mean value and standard deviation. TOS: total oxidant status; TAS: total antioxidant status; OSI: oxidative stress index, ^a^Wilcoxon's test for paired samples, ^b^a paired Student *t*-test.

**Table 3:** Analysis of levels of oxidative stress markers in groups with labor before and after 37 weeks of gestation before and after 48 hours of continuous administration of tocolytic therapy with Atosiban.

Variable		Birth before 37 weeks (*n* = 18)	Birth after 37 weeks (*n* = 35)	*P* value
TOS (*μ*mol/L)	**0 h**	24.847 ± 19.183	15.839 ± 14.135	0.060
	**48 h**	42.803 ± 34.683	25.792 ± 27.821	**0.031**
TAS (mmol/L)	**0 h**	0.937 ± 0.331	0.934 ± 0.395	0.612
	**48 h**	0.580 ± 0.336	0.565 ± 0.305	0.636
Carbonyl groups (*μ*mol/L)	**0 h**	61.670 ± 28.555	68.606 ± 30.386	0.428
	**48 h**	96.231 ± 37.574	101.104 ± 36.872	0.654

Data are presented as mean value and standard deviation. TOS: total oxidant status; TAS: total antioxidant status. Analysis of variance tests were used to examine the relationship between time of labor and levels of markers of oxidative stress.

## Data Availability

The research data used to support the findings of this study are available from the corresponding author upon request (gajzuzanna@gmail.com, zuzanna.gaj@iczmp.edu.pl).

## Abbreviations

3-NT: 3-Nitrotyrosine
OSI: Oxidative stress index
OTR: Oxytocin receptor
ROS: Reactive oxygen species
RNS: Reactive nitrogen species
TAS: Total antioxidant status
TOS: Total oxidant status.
